# Exploring the Relationship Between ESG Performance and Green Bond Issuance

**DOI:** 10.3389/fpubh.2022.897577

**Published:** 2022-05-27

**Authors:** Shanshan Wang, Derek Wang

**Affiliations:** College of Business Administration, Capital University of Economics and Business, Beijing, China

**Keywords:** green bond issuance, ESG, financial performance, environment, sustainability

## Abstract

As an important part of green financial instruments, green bonds have become increasingly popular in recent years. This study employs green bond issuance as a proxy to measure investors' recognition of a firm's sustainable activities by linking literature on ESG and financial performance and those on green bond issuance. This study innovatively creates the datasets by combining the ESG performance of Chinese listed companies with their green bond issuance from 2016 to 2020 based on the Wind and CSMAR databases and examines the relationship between the performance of ESG dimensions and green bond issuance from the perspective of listed firms in the emerging market. The results indicate that decent ESG practices not only increase the propensity in green bond issuance by listed firms but also help them issue more green bonds. More specifically, we found evidence to support this finding from every dimension of these sustainable practices. However, this study identified the negative effect of financial performance in issuing green bonds when combining the effect of ESG performance.

## Introduction

Past several decades have witnessed a profound trend that emerges in corporate sustainability, from voluntarily involving in sustainable activities to actual requirements because of both social expectations and regulatory pressure (1). In recent years, global issues such as climate warming, environmental pollution, and carbon emissions have become increasingly prominent. A great deal of firms have adapted sustainability strategies and disclosed environmental, social, and governance (ESG) information, which results in fundamental changes in business models and management theory. These changes have shifted from conventional shareholder-oriented management (2) with aims of financial performance enhancement and the shareholders' welfare maximization to stakeholder-oriented management (3), which considers all the stakeholders, including shareholders, consumers, customers, communities, and other related groups, and eliminating externalities and maximizing social value regarding ESG issues (4).

Environmental, social, and governance has obtained great attention in academia and business management in recent years (5, 6). Firms have a burden not only to maximize productivity and profitability but also to experience constant demand concerning the social and environmental impacts of their activities (7). A successful firm should implement good corporate governance practices and maintain strong relationships with society and the environment (8). As the measure of sustainable strategies, ESG performance has been widely studied in its relationship with the financial performance (FP) of firms (9–11). While some researchers found a positive effect of ESG on FP (12–14), certain researchers found negative effects (15, 16). Others concluded that there is no relationship between the ESG score and FP (17–19).

To overcome the lack of funds for the development of a green economy, sustainable financing provides a driving force for companies to seek ESG investment. As one of the important carriers of green finance, green bonds play a positive role in financing the transition to a low carbon economy (20). A green bond refers to a plain fixed income tool that can be used to finance or refinance new or existing projects accelerating the progress of economically sustainable activities (21). Green bonds build an extremely effective link between corporate finance and corporate sustainability considering their standard financial characteristics bundled with the dedication to environmental issues (22).

It is suggested that the issuance of green bonds symbolizes the attention of firms to environmental protection and green innovation, which improves the development of a low-carbon economy and green finance and builds a good social image (23). Thus, when some investors consider the contribution of firms to a green economy, they will link the financing function of green bonds with individual stocks, associating the growth of firm performance and stock price with the positive external effect of ESG practices (20). In recent years, academic researchers study green bonds in different aspects. Reboredo (24) assesses the link between the green bond market and the financial market, while Febi et al. (25) investigated the effects of the liquidity premium on the green bond yield spreads. Furthermore, Chiesa and Barua (26) examined the factors affecting green bond issuance and pricing (27).

All these studies have been focused on firms in developed countries, while the impact of this relationship on emerging country firms in China remains far from clear (28–30). The empirical evidence shown in these studies cannot be generalized to emerging markets in terms of the relevance of the value of ESG activities. It is important to emphasize that firms are significantly and systematically different from those in developed countries in terms of their social, cultural, and managerial practices (31), such as weak or dysfunctional institutions (32–34), limited state control (35), less favorable business climates, a lack of corporate governance (36, 37), higher levels of uncertainty, specifically higher corruption levels (38), and greater political risks (39). In sum, China provides an ideal research environment and unique context for us to understand the development of green bonds, and specifically for identifying the effect of ESG practices on the issuance of green bonds.

**Figure 1:**
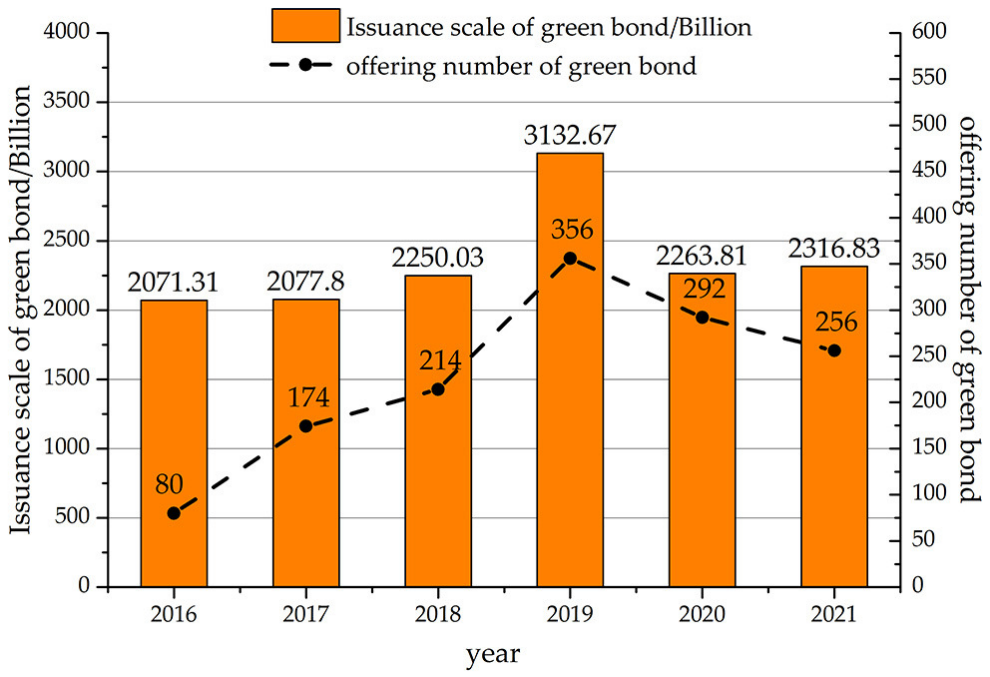
Issuance scale of green bonds in China.

According to the World Bank's WDI database, China is the largest carbon emitter and developing economy. Cheap labor and heavy investment contributed to China's past economic growth, but a huge environmental price was also paid for this development. The financial market is adjusted by the Chinese government to relieve the contradiction between economic growth and environmental protection (40). China's SRI investment market is currently in the early stages of rapid development (41). The green bond market is one of the major financial innovations promoted by the central government. As shown in Figure 1, the issuance of green bonds in China has been booming in recent years (20). It achieves the highest amount ever in 2019, although the issuance retreats in 2020 and 2021 due to the effect of the pandemic.

This research aimed to explore the relationship between ESG performance and green bond issuance. It is achieved by two stages of analysis in this study, which are to investigate whether a listed firm with good ESG performance issues green bonds and to examine the volumes of green bonds issuance by the listed firms who provide better ESG practices. Our result confirms that good ESG performance and practices lead to more green bond issuance, even when considering their sub-factors from the environment, society, and governance dimensions. These factors enable investors to identify a responsible and sustainable firm.

Based on the Wind and CSMAR databases, this research builds a dataset combining the ESG performance of Chinese listed companies with green bond issuance between 2016 and 2020 to examine the relationship between green bond issuance and ESG dimensions. In addition, probit models are used to identify variables that impact the issuance of green bonds.

This study makes several key contributions. First, only a few studies directly established the relationship between ESG practices and investment recognition, and there is no research on the impact of ESG practices on green bond issuance. This study innovatively constructs the relationship between ESG practices of Chinese listed companies and investment recognition and discusses in detail how ESG practices affect green bond issuance. This study does not treat the green bond as a simple debt but as a recognition of the sustainable and responsible firm by investors. It employs the issuance of the green bond as a proxy of such recognition, which innovatively builds a link between the green bond and ESG practices. This study neither observed green bond from the perspective of pure debt nor examined the ESG from the perspective of its impact on financial performance. It combines both aspects and extends the literature on green bonds and ESG study. In doing so, this study incorporates the ESG scores and financial performance into the model of impact on the issuance of green bonds by creating an unprecedented dataset, which probes the roles of ESG practices in green investment.

Second, although the investment philosophy of ESG is gradually accepted, most of the related products are based on ESG overall scores and indexes related to the environment (E), but study on indexes related to society (S) and corporate governance (G) are relatively scarce. At present, there is a lack of research on specific indicators in the ESG evaluation system and green bond issuance. This article not only illustrates the effect of ESG scores on green investment as a whole indicator but also examines environmental (E), social (S), and governance (G) separately to determine accurately the relationship of each sub-factor to green investment in China. In terms of the three dimensions, we used specific practices to be proxies of the three sub-factors, which makes the indicators more detailed and more inclined. Different institutions have different scoring frameworks and evaluation criteria for companies' ESG practices. The use of processed “indirect data” evaluated by institutions cannot fully reflect the company's real situation to a certain extent, and it is impossible to explore the specific paths through which different practices affect investment decisions, and affect the richness of research results. This study uses the “direct data” on ESG practices disclosed in the annual reports of listed companies to more truly and accurately establish the relationship between them and investment recognition. By applying resource-based views, the analysis of the influence of ESG scores and individualized effects of each sub-factor (E–S–G) on green investment contributes to the literature on multinational firms (42).

Third, there is a great deal of controversy on the practice of socially responsible investment in investment management, including financial performance, and no consensus has yet been reached. This article explores the moderating effect of financial performance in green investment. It reveals the contradictive relationship between ESG performance and financial performance, especially its effect on influencing the issuance of green bonds. This creatively echoes the argument about the ESG investment's negative effect on financial performance.

Fourth, previous studies have mainly focused on the effect of ESG in developed countries. Most of the existing ESG studies use developed markets as samples, and there is a lack of research on emerging markets such as China (9). This study focuses on firms in the emerging market. Although studies about emerging economies are in very recent literature (30, 38), few empirical ones have been performed on ESG dimensions in green investment. The research on China's ESG evaluation system is still in the theoretical stage, and the concept of ESG investment has not been widely popularized and applied. Since this relationship has not been directly explored in the context of China, these findings fill an important gap in the field.

The remainder of this article is organized as follows. First, this article summarizes relevant literature on the relationship between ESG with financial performance and green bond issuance activities. On the basis of previous studies, this article proposes hypotheses from both ESG and its sub-factor (E–S–G) dimensions. Then, in the data and methods section, this article describes the sample, the variables, and models used in this study, including the probit model and other regression models. Furthermore, this article explains the empirical results and discusses the result from both theoretical and managerial views. Finally, the last section concludes and points out the limitations.

## Literature Review and Hypotheses

### ESG and Green Bond Issuance

It is argued that if a firm uses its resources more sustainably, it will generate some clear positive outcomes from a view of economic efficiency. Some studies identified that, for firms involving well-developed environmental management systems, the debt financing cost is lower compared with their rivals (43). In particular, recent literature revealed that firms with corporate social responsibility (CSR) policies and high CSR performances can issue bonds at a lower cost, and thus investors have a large investment pool deduced by such a higher market evaluation (44). ESG has evolved from CSR as the development of firm sustainable strategies. Therefore, a higher sustainability performance can lead to a lower-cost equity capital (45). This results in a high chance of green bond issuance in good ESG practices, which is shown in the below hypothesis.

Hypothesis 1a: The firms with good ESG practices are more likely to issue the green bond.

It is shown that firm solvency and their ratings are positively related to environmental practices, consequently, implying their low risk in potential legal, regulatory, and reputational costs (46). Firms without effective sustainable practices will potentially experience expensive fines and strong resistance from stakeholders, which can increase their default risk and liabilities (47). Polbennikov et al. (48) corroborated that bonds with higher ESG scores have higher returns. Furthermore, the issuer of the green bond with green certification and sufficient information disclosure will decrease the screening costs of investors and improve the confidence of investors in green bonds. Thus, the high bond premium and low company financing costs resulting from sustainable practices stimulate the issuance of green bond (20). This is shown in the hypothesis 1b.

Hypothesis 1b: The good ESG practices will facilitate firms to issue more green bonds.

### The Moderation of Financial Performance

To date, a great deal of empirical literature has examined the relationship between corporate financial performance (CFP) and corporate sustainability to explore the implications of stakeholder-oriented management for CFP (14, 19, 49). According to the Porter hypotheses (50), corporate social responsibility (CSR) activities, especially environmental activities, create excess turnover which can cover the additional costs, thus an appropriate sustainable strategy can improve CFP. Most of the empirical studies display a positive relationship between CFP and CSR (19, 51, 52). However, agency problems and inefficient resource allocation could generate additional costs in sustainable activities, which results in a disadvantage for firms in the free and competitive market (2, 53). Some empirical studies with negative relationships were documented (54). For instance, Lee and Faff (55) found that ESG investment worsens CFP.

In addition to positive and negative relationships, a neutral relationship has also been found between CFP and CSR (56). However, according to Barnett and Salomon (57), the relationship between CFP and CSR is neither strictly positive nor strictly negative. They found an inverted U-shaped relationship between CFP and corporate environmental performance, which exhibits evidence for the nonlinear relationship (58, 59).

According to the traditional neoclassical approach, investing in ESG activities brings additional costs for a firm (60), which impacts CFP. For instance, investments in reducing emissions or improving the use of natural resources are excessive (61, 62). In a production process, the cost of considering its effect on the environment, clear emissions reduction, noise control, or waste management policies is high. When these firms decide not to invest in environmental initiatives, they could avoid economic resources being compromised, and their performance increases in the case that environmental goals are not priorities for them. Thus, even if good ESG practices encourage green bond issuance, this trend will be compromised by the moderating effect of potentially good financial performance. This deduces hypothesis 2.

Hypothesis 2: When considering the firm's financial performance, it will negatively interfere with the positive effect of ESG practices on green bond issuance.

### The Practices in Three Dimensions of ESG

Since the ESG score is weighted on a company's performance in the environmental (E), social (S), and governance (G) sub-factors, a company possibly involves in individual E, S and G activities at different levels (63). The practices developed from individual one of these three dimensions could improve the financial value for some firms but undermine it for other firms (64). There is no consensus on the actual effect of individual ESG on green investment. Therefore, to obtain a better understanding the impact of ESG activities on green investment, a more detailed analysis of the sub-factors may be necessary.

#### Environmental Dimension

In terms of the environmental dimension of ESG activities, it is argued that environmental regulations result in an additional cost for the company that decreases profitability and efficiency. In contrast, according to the Porter hypothesis, the strict but flexible environmental regulation may provide companies with incentives to innovate technologically or managerially. This improves efficiency that neutralizes the additional costs and grows more revenue eventually (4). However, it is still questioned whether environmental regulations can generate additional revenue and higher corporate efficiency by offsetting the excess cost (65). Additionally, firms often show inconsistent words and actions in conforming to environmental policies (66). Empirically, the strict environmental standards will stimulate higher market value than that of less strict regulations (67). In a high-growth industry, profitability is positively linked with environmental performance (68). Such profitability impacts the recognition of these firms by investors. Thus, a firm's environmental performance positively affects the issuance of green bonds issued by the firm (21). Unfriendly environmental activities will negatively impact the issuance of green bonds. According to the previous studies discussed above, we propose hypothesis 3a.

Hypothesis 3a: Unfriendly environmental activities are negatively related to the issuance of green bonds.

#### Social Dimension

In terms of the social dimension of ESG activities, social activities show controversial relationships with financial performance. On the one hand, Brammer and Millington (69) supported a positive relationship between good corporate social performance, such as charitable giving, and financial performance in the long term. Besides, reputation, brands, and large quantities of natural resources could strengthen these benefits in some sectors (70). However, social activities share the same concerns regarding additional costs with environmental activities. Costs come along with practicing social activities, for example, having a health and safety policy. It is also argued that providing employees with CSR training cannot contribute to the financial performance. These controversial arguments support that if the additional costs can be addressed by the benefits, social activities, including corporate reputation enhancement and able employees' attraction, will improve profits and efficiency (4). These firms' social practices will be further positively related to the issuance of green bonds (21).

Hypothesis 3b: Social activities that attract capable employees are positively related to the issuance of green bonds.

#### Governance Dimension

In terms of the governance dimension of ESG activities, a board structure is focused on several aspects to explore the firm's performance. Zhu et al. (71) found that a firm's value tends to be improved if the firm sets up the structure of independent directors. In certain industries, such as the banking industry, good corporate governance has a positive effect on financial performance (72). The reputation and status in society of independent directors ensure that a firm's attention is paid more to environmental opportunities and development in corporate innovations (73). The existence of independent executive directors on the board is beneficial to the control over the quality of information disclosure (74). Therefore, an audit and/or supervisory committee composed of independent directors will ensure such practices and make the image of a responsible company to investors (75). Polbennikov et al. (48) identified the positive relationship between ESG bond scores, including individual governance score and bond performances.

From another aspect, shareholders have less control over management (76) and less communication with executives (77) in firms with relatively dispersed ownership. The less diversified stakes may provide more assurance in engaging sustainable practices (78). Thus, the shareholding of the large shareholders could force management to disclose more information related to environmental responsibility and increase the success of green bond issuance (75).

According to the discussion above, several activities regarding global sustainable strategies and board composition are positively associated with firm performance and efficiency (4). Following the previous studies, we further added global sustainable governance principles as a factor in our analytical model to investigate how governance activities are linked with green bond issuance. We propose the following hypothesis.

Hypothesis 3c: Governance activities that involve an independent and diversified board of directors are positively related to green bond issuance.

## Data and Methods

### Data Collection

The article employs the green bonds issued by companies listed in the Chinese stock market between 2016 and 2020 in the Wind database. We creatively combined the information on green bond issuance and financial data from the CSMAR database. Previous studies (20, 75) examined issues on ESG performance or green bond issuance using the Wind and CSMAR databases, which provide a theoretical basis for this research. In addition, Wind and CSMAR databases are vital in terms of their comprehensive data. The selected period comes primarily for two reasons. First, China began issuing green bonds in 2015, but data are available from 2016. Second, green bond issuance before 2021 is essentially unaffected by the COVID-19 pandemic. As a result, this research selected data from 2016 to 2020.

In the remainder of the analysis, we limit the sample to the green bonds of these listed companies. After excluding unlisted companies that issue green bonds and companies listed on the Hong Kong Stock Exchange and other overseas stock exchanges, a total of 94 samples of listed companies remain. In addition, this study considers industry factors, and we divided them into 19 industries according to the China Securities Regulatory Commission (SEC)'s industry classification.

### Variables and Model

#### Dependent Variable

As shown in Table 1, the dependent variable is green bond issuance. In the first stage of analysis, whether a listed firm issues a green bond is introduced as a dummy to construct a probit model. Furthermore, the second stage of analysis investigates the volume of green bond issuance in these listed firms to identify the factors of investors recognizing a responsible and sustainable firm.

**Table 1:** Variable description.

**Type**	**Variable name**	**Variable symbol**	**Variable description**
Dependent variable	Green bond dummy	D_greenbond	The likelihood of green bond issuance
	Green loan volume	Greenloan3	The volume of green bond issuance
	ESG scores	esg_huazheng	ESG rating of Shanghai Huazheng index information service Co., Ltd
	Environmental dimension	ln_water ln_greenhouse_gas	Total water consumption Total greenhouse gas emissions
	Social dimension	ln_employee	Total number of employees
Independent variable	Governance dimension	Supervisory_chair	Is there a chairman of the supervisory board
		top10_shareholder	Stocks held by the top 10 major stockholders/all stocks
	Financial performance	roa	Return on Assets, which is net margin/total assets
	Leverage	gearingrate	Asset-liability ratio, which is liabilities/total assets
	Firm size	ln_total_asset	Natural logarithm of total assets (ln)
	Firm IPO age	ipo_age	The number of years since its first IPO
Control variable	Firm ownership	soe	Dummy variable, 1 for state-owned listed companies, or 0
	Firm growth	Sales_growth	Increase rate of business revenue, which is amount of operating income for the current year-amount of operating income for the same period of the previous year

#### Independent Variables

In the third-party independent rating in the Wind database, compared with several other ESG rating scores, Huazheng's ESG rating score^1^ covers more comprehensive data. Therefore, this study used the ESG rating scores retrieved from the Wind database as independent variables. The total ESG score, which is called the Huazheng ESG rating score, can be classified as an added value of CSR performance for the three subgroups (E, S, and G). Values range from 0 to 9, with 9 as the highest score.

This study next investigated the effect of a firm's financial performance (FP). Return on Assets (ROA) is used in this article as a proxy for the firm's FP. ROA is widely utilized in the literature as a proxy to assess the impacts of ESG on FP (79–81). ROA is defined as the net income's ratio to total assets and focuses on how a company's earnings respond to different managerial policies and the relative efficiency of asset utilization (55). In the second stage, the interaction term between financial performance and ESG scores is used to assess the moderating role of FP in green bond issuance.

This article also analyses the impacts of the three E, S, and G score components separately: the environmental dimension is measured by water usage and greenhouse gas emission; the social dimension is measured by the number of employees; and the governance dimension is measured by the shareholding stake of top 10 shareholders and whether there is a chairman of the supervisory committee.

#### Control Variables

To exclude the impact of other factors on the issuance of green bonds, several control variables were introduced into the regression models.

- Firm ownership: Since the state-owned economy is the dominant part of the Chinese economy, state-owned firms bear greater social responsibilities and will also bear more environmental responsibilities (82). Hence, we used firm ownership as a control variable and measured it as a dummy variable (1 for state-owned enterprises and 0 otherwise) (75).
- Firm IPO age: Firms with longer listing time have a better reporting structure (83) and have a higher awareness of great environmental pressure (84). Therefore, the longer the firms' listing time, the higher the degree of corporate environmental responsibility disclosure. Consequently, we used firm IPO age as a control variable and measured it with the number of years since its first IPO (75).
- Firm size: Larger firms obtain more public attention and subjected to greater political and regulatory pressures from external stakeholders (85), so large firms tend to disclose more information to illustrate that their actions are legitimate and consistent with good corporate citizenship (76, 78, 86). Consequently, we used the firm size as a control variable and measured it with the logarithm of total assets (75).
- Leverage: Firms with low financial leverage have more resources and the intention to disclose environmental responsibility information (78, 86) to ensure that the market participants properly evaluate their financial risks (87). Hence, we estimated that there is an inverse relationship between corporate financial leverage and green bond issuance. Consequently, we used firm leverage as a control variable and measured it with the ratio of total debt divided by total assets (75).

#### Model Setting

This study first used a probit model to explore what factors impact the likelihood of green bond issuance. Probit models can be used for modeling the relationship between one or more numerical or categorical predictor variables and a categorical outcome. For the probit model, the following relationship is assumed:


(1) $$\begin{array}{r} \text{P}(\text{Y }=\;1|{\text{x}}_{1},\;.\;.\;.\;,{\text{ x}}_{\text{k}})\;=\Phi \;(\beta_{0}\;+\;\beta_{1}{\text{ x}}_{1}\;+\;.\;.\;.\;+\;\beta_{\text{n}}{\text{ x}}_{\text{n}}) \end{array}$$

where Φ denotes the distribution function of the standard normal distribution, and it also transforms the regression into the interval (0, 1). The regression coefficients of the probit model are effects on a cumulative normal function of the probabilities that Y = 1 (i.e., the probability that a firm issues a green bond). As such, its metric can easily be understood as a standard normal score. Using this, the coefficients can be interpreted directly.

The parameters of the probit model need to be computed *via* a non-linear method such as maximum likelihood estimates (MLE) or nonlinear optimization techniques. These parameters cannot be solved *via* ordinary least squares (OLS). The probability of the event y being observed is then computed from the inverse of the normal distribution. This is:


(2) $$\begin{array}{r} \text{prob}(\text{y})\;=\text{ F}-1(\text{z}) \end{array}$$

Our first stage model is expressed as follows:


(3) $$\begin{array}{r} \text{D}\text{\_}{\text{greenbond}}_{\text{i}\text{t}}\;=\;\alpha_{0}\;+\;\alpha_{1}^{*}\;\text{esg}\_{\text{huazheng}}_{\text{i}\text{t}}\;+\;\alpha_{2}^{*}\;{\text{r}o\text{a}}_{\text{i}\text{t}}\\ +\alpha_{3}^{*}\;{\text{X}}_{\text{i}\text{t}}+\varepsilon_{\text{i}\text{t}} \end{array}$$

Where X_it_ denotes the vector of control variables, including Leverage, Firm size, Firm IPO age, Firm ownership, and Firm growth. ε_it_ is the error term.

In the second stage, our models are run in the OLS regressions. They are expressed as follows:


(4) $$\begin{array}{r} \begin{array}{l} \text{greenloan}3_{\text{it}}\;=\;\beta_{0}\;+\;\beta_{1}^{*}\text{esg}\_{\text{huazheng}}_{\text{it}}+\beta_{2}^{*}{\text{ roa}}_{\text{it}}\;+\;\beta_{3}\\ {\;}^{*}\text{esg}\_\text{roa }+\;\beta_{4}^{*}{\text{ X}}_{\text{it}}\;+\mu_{it} \end{array} \end{array}$$

(5) $$\begin{array}{r} {\text{greenloan}3}_{\text{i}\text{t}}\;=\;\delta_{0}+\delta_{1}^{*}\;{\text{E}}_{\text{i}\text{t}}\;+\delta_{2}^{*}\;{\text{S}}_{\text{i}\text{t}}\;+\delta_{3}^{*}\;{\text{G}}_{\text{i}\text{t}}\;+\;\delta_{4}^{*}\;{\text{r}o\text{a}}_{\text{i}\text{t}}\\ +\;\delta_{5}^{*}\;{\text{X}}_{\text{i}\text{t}}\;+\sigma_{\text{i}\text{t}} \end{array}$$

Where X_it_ denotes the vector of control variables, and μ_it_ and σ_it_ are the error terms. esg_roa is the interaction term between ESG score and financial performance. E_it_, S_it_, and G_it_ are environmental, social, and governance dimensions of ESG practices, which are measured by ln_water, ln_greenhouse_gas, ln_employee, top10_shareholder, and supervisory_chair, respectively. We have attempted all combinations of variables within these dimensions and reported the representative models in the Results section.

Ordinary least squares (OLS) regression is a useful, easily interpretable statistical method. However, in regression analysis, the presence of outliers in the dataset can strongly distort the classical least-squares estimator and lead to unreliable results. For instance, when running an OLS regression, it can at times be highly affected by a few records in the dataset and can then yield results that do not accurately reflect the relationship between the explained variable and the explanatory variables seen in the rest of the records. To address this, several robust-to-outliers methods have been proposed in the statistical literature. Robust regression offers an alternative to OLS regression that is less sensitive to outliers and still defines a linear relationship between the outcome and the predictors. As such, we adopted the robust regression in the second-stage analysis.

## Analysis and Results

### Descriptive Statistics

Descriptive statistics are provided in Tables 2, 3 along with correlation coefficients. The sample size of greenloan 3 is 300 with a mean of 42.147 and a standard deviation of 130.173. The minimum and maximum of greenloan 3 are 0 and 1,000, respectively. The mean of greenloan 3 is rather high, indicating that it has a large issuance, and the high standard deviation means that it has high issuance dispersion, that is, the issuance of greenloan 3 is uneven, presenting a very changing curve.

**Table 2:** Descriptive statistics.

**Variable**	**Obs**	**Mean**	**Std. Dev**.	**Min**	**Max**
Greenloan 3	300	42.147	130.173	0	1,000
esg_huazheng	264	7.273	1.141	4	9
ln_water	54	13.749	3.707	9.572	23.706
ln_greenhouse_gas	29	12.345	3.673	8.387	19.633
top10_shareholder	300	63.534	16.596	20.580	99.828
supervisory_chair	300	0.573	0.495	0	1
ln_employee	199	9.135	1.555	3.989	13.116
roa	210	5.407	3.407	−13.115	15.078
gearingrate	300	70.846	17.387	18.697	95.020
ln_total_asset	300	25.401	2.091	21.284	30.934
ipo_age	300	11.567	8.359	−4	28
soe	300	0.650	0.478	0	1
sales_growth	300	13.981	21.577	−47.461	119.763

*Greenloan 3, Green loan volume; esg_huazheng, ESG scores; ln_water, Environmental dimension; ln_greenhouse_gas, Environmental dimension; top10_shareholder, Governance dimension; supervisory_chair, Governance dimension; ln_employee, Social dimension; roa, Financial performance; gearingrate, Leverage; ln_total_asset, Firm size; ipo_age, Firm IPO age; soe, Firm ownership; sales_growth, Firm growth*.

**Table 3:** Correlation between variables.

		**1**	**2**	**3**	**4**	**5**	**6**	**7**	**8**	**9**	**10**	**11**	**12**	**13**
1	Greenloan 3	1												
2	esg_huazheng	−0.010	1											
3	ln_water	−0.215	0.331^**^	1										
4	ln_greenhouse_gas	−0.131	0.285	0.935^***^	1									
5	top10_shareholder	−0.080	0.256^***^	0.352^***^	0.376^**^	1								
6	supervisory_chair	0.048	−0.016	0.284^**^	0.235	−0.063	1							
7	ln_employee	0.261^***^	0.175^**^	0.326^**^	0.317^*^	0.221^***^	−0.137^*^	1						
8	roa	−0.082	0.149^**^	−0.296	−0.363	0.153^**^	0.033	−0.260^***^	1					
9	gearingrate	0.266^***^	−0.118^*^	−0.539^***^	−0.509^***^	−0.068	−0.082	0.446^***^	−0.441^***^	1				
10	ln_total_asset	0.400^***^	0.145^**^	−0.296^**^	−0.265	0.157^***^	−0.011	0.686^***^	−0.273^***^	0.772^***^	1			
11	ipo_age	0.015	0.218^***^	0.384^***^	0.616^***^	0.101^*^	0.159^***^	0.081	0.013	−0.421^***^	−0.072	1		
12	soe	0.139^**^	0.162^***^	0.126	0.176	0.207^***^	0.116^**^	0.078	0.158^**^	−0.095	0.179^***^	0.284^***^	1	
13	sales_growth	−0.067	0.034	−0.073	−0.057	−0.059	0.098^*^	−0.090	0.243^***^	−0.039	−0.127^**^	−0.163^***^	−0.152^***^	1

*Greenloan 3, Green loan volume; esg_huazheng, ESG scores; ln_water, Environmental dimension; ln_greenhouse_gas, Environmental dimension; top10_shareholder, Governance dimension; supervisory_chair, Governance dimension; ln_employee, Social dimension; roa, Financial performance; gearingrate, Leverage; ln_total_asset, Firm size; ipo_age, Firm IPO age; soe, Firm ownership; sales_growth, Firm growth*.

*** * = statistically significant at 1%*,

** *= 5%, and*

* *= 10%*.

The sample size of esg_huazheng is 264 with a mean of 7.273 and a standard deviation of 1.141. The minimum and maximum of esg_huazheng are 4 and 9, respectively. The mean of esg_huazheng is relatively high, indicating that firms perform better sustainable practices generally, and the low standard deviation means that it has even score distribution and low dispersion. Furthermore, according to its minimum and maximum, it fluctuates within small ranges from 4 to 9.

### Probit Model and Marginal Effects

Table 4 illustrates the marked differences between green bond issuance and ESG rating score between listed public firms with issuing green bonds and those without issuing green bonds. Notably, the ESG activities appear important in explaining the performance of green bonds. However, we seek to move the debate on the role of Huazheng ESG rating score in explaining the likelihood of green bond issuance within listed companies by controlling for other financial information. The significant and positive sign of Huazheng ESG rating score shows that public firms with good sustainable practices are more likely to successfully issue green bonds, which confirms hypothesis 1. It shows that the probability of green bond issuance will increase 0.073% if the listed firm improves its Huazheng ESG score by 1 grade.

**Table 4:** The likelihood of green bond issuance within listed companies.

	**Probit model**	**Marginal effects**
	**D_greenbond**	**D_greenbond**
**D_greenbond**		
esg_huazheng	0.042^**^	0.001^**^
	(0.021)	(0.000)
roa	0.001	0.000
	(0.000)	(0.000)
gearingrate	0.000^**^	0.000^**^
	(0.000)	(0.000)
ln_total_asset	0.313^***^	0.005^***^
	(0.016)	(0.000)
ipo_age	−0.014^***^	−0.000^***^
	(0.003)	(0.000)
soe	0.213^***^	0.004^***^
	(0.050)	(0.001)
sales_growth	−0.000	−0.000
	(0.000)	(0.000)
Pseudo R2	0.162	
No. of observations	29,241	29,241

*D_greenbond, Green bond dummy; esg_huazheng, ESG scores; roa, Financial performance; gearingrate, Leverage; ln_total_asset, Firm size; ipo_age, Firm IPO age; soe, Firm ownership; sales_growth, Firm growth*.

*** *= statistically significant at 1%*,

** = 5%, and *= 10%.

Table 5 lists the impact of ESG practices on green bond issuance and particularly the roles of individual dimensions of ESG. Model 1 is the baseline model in which we explored the impact of Huazheng ESG rating score on green bond issuance. The significant and positive sign of the coefficient represents that the higher the ESG score, the larger the volume of green bond that is successfully issued. This confirms hypothesis 1b. In this model, ROA shows negative and significant sign, which implies that a firm's good FP will negatively affect the green bond issuance. This can be explained that less input into ESG practices is often associated with cost saving, which results in good FP in the short term. Thus, it reduces the green bond issuance. The negative and significant sign of the gearing ratio indicates that financially vulnerable firm usually issues fewer green bonds because investors would be concerned about its high proportion of debt and operational risk. In terms of firm size, the positive and significant sign of total asset elaborates that a large firm can issue more green bonds.

**Table 5:** The volume of green bond issuance within listed companies.

	**(1)**	**(2)**	**(3)**	**(4)**	**(5)**
	**Greenloan 3**	**Greenloan 3**	**Greenloan 3**	**Greenloan 3**	**Greenloan 3**
esg_huazheng	1.792^**^	4.142^***^			
	(0.766)	(1.468)			
roa	−0.488^*^	2.742^**^	−0.209	−0.082	−2.588^*^
	(0.291)	(1.290)	(0.720)	(0.971)	(1.092)
gearingrate	−0.206^*^	−0.317^**^	−1.127^***^	−1.350^***^	−1.102^**^
	(0.121)	(0.134)	(0.365)	(0.442)	(0.384)
ln_total_asset	3.172^*^	3.278^**^	5.206	10.200	−33.570
	(1.638)	(1.636)	(6.174)	(7.452)	(24.490)
ipo_age	0.238	0.191	−1.459^*^	−1.699^*^	−1.947
	(0.226)	(0.231)	(0.767)	(0.894)	(1.866)
soe	−1.700	−2.035	−10.920^*^	−8.008	−38.740^**^
	(2.961)	(3.030)	(5.245)	(6.183)	(11.960)
sales_growth	0.015	−0.005	−0.008	−0.029	0.095
	(0.044)	(0.046)	(0.095)	(0.113)	(0.091)
esg_roa		−0.500** (0.217)			
ln_water			−3.486*** (0.496)	−3.514*** (0.584)	
top10_shareholder			0.229** (0.088)		
ln_employee			17.180*** (2.791)	16.410*** (3.496)	14.740** (4.855)
supervisory_chair				−4.535 (5.102)	9.630* (4.380)
ln_greenhouse_gas					6.896 (3.507)
cons	−62.650^**^	−71.820^**^	−140.900	−223.700^*^	753.500
	(30.440)	(30.700)	(100.300)	(118.400)	(484.500)
*R* ^2^	0.117	0.133	0.972	0.962	0.999
*N*	199	199	25	25	14

*Greenloan 3, Green loan volume; esg_huazheng, ESG scores; ln_water, Environmental dimension; ln_greenhouse_gas, Environmental dimension; top10_shareholder, Governance dimension; supervisory_chair, Governance dimension; ln_employee, Social dimension; roa, Financial performance; gearingrate, Leverage; ln_total_asset, Firm size; ipo_age, Firm IPO age; soe, Firm ownership; sales_growth, Firm growth*.

*** *= statistically significant at 1%*,

** *= 5%, and*

* *= 10%*.

Model 2 adds the interaction term between ESG score and ROA based on model 1 to probe the moderating role of financial performance. In model 2, this interaction term shows a negative and significant sign while ROA's sign becomes positive and significant. It suggests that FP includes two elements in affecting green bond issuance which are the roles of itself and its moderating effect. Good FP, *per se*, increases green bond issuance, but it shows a negative effect on issuance when it moderates ESG practices. Furthermore, the interaction term distinguishes the roles of these two elements, so ROA displays its positive effect on its own and the interaction term represents the negative role of its moderating effect on ESG activities in model 2. This confirms hypothesis 2 and not only supports the previous literature on the negative effect of financial performance but also creatively decomposes this negative effect. Except for these two variables, model 2 reports the same sign and significance as model 1 in terms of ESG score and other control variables. This confirms the baseline model.

Models 3, 4, and 5 examine the individual dimensions of ESG activities separately. In model 3, water usage shows a negative and significant sign, which indicates that the bad environmental practice will hinder the green bond issuance. This supports hypothesis 3a. The shareholding of the top 10 shareholders reports a positive and significant sign. It implies that the good governance structure increases the successful issuance of green bonds because concentrated shareholding entitles investors more power to make management comply with the ESG requirement. This supports hypothesis 3b. In terms of the social dimension, the positive and significant sign of employee number exhibits that more employment will increase green bond issuance because employment encouragement convinces investors that the company is responsible. This supports hypothesis 3c.

Particularly, model 3 reports negative and significant signs of IPO age and state-owned firm. Although firms with a long history since IPO or state-owned status often show better practices from the perspective of information disclosure and ESG activities, they have more channels to obtain finance other than green bonds. Therefore, green bond issuance constitutes a small proportion of their debt finance. In addition, the longer firm is listed, the less it issues green bond, and particularly when it is state-owned. The gearing ratio is consistent with models 1 and 2.

Model 4 generally confirms model 3 in terms of environmental and social dimensions of ESG, gearing ratio, and IPO age. Model 5 replaces water usage and shareholding of top 10 shareholders with greenhouse gas emission and chair of the supervisory committee based on environmental and governance dimensions of ESG, respectively. It shows that good governance structure and social responsibility will obtain more recognition of sustainable firms from investors, and thus issue successfully more green bonds. However, there is no evidence of increased green bond issuance from the environmental consideration. When considering individual dimensions of ESG, a firm's financial performance expresses a negative effect, which is consistent with model 1.

## Discussion

To date, research on the relationship between the performance of ESG and green bond issuance remains a less-explored area in emerging markets. In particular, less attention has been paid to the context of China. Van Duuren et al. (88) found that institutional investors focus on corporate governance aspects of ESG, while individual investors focus more on environmental aspects. Compared with most empirical studies in Chinese literature, which are based on a single perspective among environmental responsibility, social responsibility, or corporate governance, this study analyzed these three factors as a whole based on the perspective of ESG rating by selecting the representative practices of the three dimensions. We addressed this gap in the research by studying the relationship between the performance of ESG dimensions and green bond issuance from the perspective of listed firms in the emerging market. Our empirical results indicate that ESG scores are positively associated with the likelihood of green bond issuance according to a probit model regression. The good ESG performance also helps listed firms achieve large issuance of green bonds. More specifically, we found the evidence to support this finding from every dimension of these sustainable practices.

Some investors doubt that ESG performance factors can help companies manage risk, provide profits or avoid minefields. They believe that ESG investment will increase the cost of enterprises, which will lead to lower profits, but ignore the hidden value of the ESG investment concept to help enterprises avoid risks and accumulate reputation to attract talents. The ESG system can avoid risks by guiding and regulating the microscopic behavior of enterprises and reducing negative events in the enterprise environment and social perspectives. This research demonstrates the relationship between ESG performance and green bond issuance through empirical evidence, helps investors to correctly understand ESG investment, and changes the previous concept that “ESG investment is just a sentimental investment,” so that it can actively incorporate ESG performance into the investment and decision-making process (89).

Furthermore, our study differs from previous literature whose findings focus on the negative relevance of relations between ESG and financial performance, that is, firms with the best ESG scores tend to be less profitable. When considering the effect of financial performance on green bond issuance, it shows two aspects of its role. On the one hand, the good financial performance, *per se*, which indicates creditworthiness, promotes green bond issuance. On the other hand, its destructive effect will display by combining with ESG. This occurs because costs related to the implementation of ESG initiatives influence a firm's financial performance in short term. Investments in ESG may nibble a firm's cash flow and divert resources required for its operation. Thus, the interactive effect will reduce the volume of successful green bond issuance. Through the analysis above, this study makes companies fundamentally aware of the importance of ESG investing. From the perspective of long-term development, investors will gradually prefer to invest in companies with good ESG ratings and use ESG ratings to screen and avoid negative companies (90). That is, companies with better ESG performance will gain greater support in the market, which will force companies to increase their emphasis on the environment, society, and corporate governance, and ultimately have a positive impact on the sustainability of China's economic green transformation.

Our study has significant implications for managers and policy makers. From a managerial point of view, the results suggest that managers and executives should pay attention to issues in the societies and environment during their operation because a firm's strategy integrating ESG considerations allows them to have greater reputation, accountability, and credibility. This encourages managers to deploy efforts and resources toward long-lasting ESG practices to achieve the company's legitimacy in local markets. Meanwhile, managers should convert their cognition of ESG to be an investment rather than an expense. Such commitments as addressing the different social and environmental needs, institutional requirements and expectations of stakeholders in the different markets will enhance their competitive power and consequently improve their long-term financial performance. Additionally, governmental and regulatory powers at the national and international levels should encourage firms to apply best ESG practices, which attracts more firms to formulate and implement advanced and responsible environmental, social, and governance initiatives.

As the development of socially responsible investing in emerging markets lags behind developed economies, emerging market investors' awareness of ESG is also relatively backward. The investment management practice of long-term funds in China still adopts the performance evaluation methods of short-term investment. Investors value short-term interests so that the investment advantages of long-term funds cannot be effectively utilized. The purpose and practical significance of this research is to comprehensively study the practice of ESG-responsible investment in China, change the operating goals of listed companies, and guide them to the long-term goal of emphasizing social value and sustainable development. This will help value-oriented institutional investors establish and develop a practical approach to ESG-responsible investment (91).

## Conclusions

This study examined the green bond market in China, which has attracted increasing attention in recent years. Data for Chinese listed companies, which have been issuing green bonds since 2016, are analyzed to determine the impact of the issuing companies' ESG practices on green bond issuance. The relationship between ESG and a firm's financial performance has been widely discussed, but we sought to examine investors' recognition of the firm's ESG activities, that is, responsible and sustainable companies. To reveal this veil, we employed green bond issuance as a proxy to measure investors' recognition of the firm's sustainable activities by linking literature on ESG and financial performance and those on green bond issuance. In doing so, we innovatively created the datasets by combining the ESG performance of Chinese listed companies with their green bond issuance from 2016 to 2020. The results indicated that decent ESG practices not only increase the propensity in green bond issuance by listed firms but also help them issue more green bonds. Given that ESG scores are determined by many factors, each of which may have a different impact on green bond issuance, we analyzed the individual effects of the E, S, and G dimensions, respectively. They all confirm the previous hypotheses. However, this study identified the negative effect of financial performance in issuing green bonds when combining the effect of ESG performance. This reveals that some listed firms do not place social or environmental goals as priorities in their corporate strategies in the short term, which are consistent with the findings of prior studies. The research results provide experience and reference for the continuous progress of Chinese listed companies and enhance the attention of listed companies on ESG practices. Implementing ESG practices promotes corporate growth and achieves a win–win situation, thereby promoting the sustainable growth of Chinese listed companies and the green transformation of the national economy.

Our study has several limitations. First, the ESG performance and its three dimensions data considered in our sample originate from only listed companies in mainland China due to the availability of data. In future research, it would be interesting to include other firms from China and other listed firms from other stock exchange markets for comparison. Second, the data used for the ESG performance have a global score based on secondary data. Although the variable has been widely used in the recent International Business literature and is treated to facilitate statistical analyses, the score assigned to each variable is not free of subjective influences, which may decrease the validity of our results. For comparison, we used objective secondary data for E, S, and G dimensions, respectively, to address this subjectivity issue. Future studies could choose other alternative and innovative measures of ESG performance (i.e., information derived from other secondary databases such as Sustainalytics and KLD, and information obtained through questionnaires and interviews). Third, it can be seen that the green bond market in China emerged in recent years, thus our study could only use data starting from 2016 to 2020. The short period and relatively small sample size could affect the accuracy of some results. The study of green finance and ESG is an interesting and novel topic in China. Therefore, when there is more perfect data for the green bond in the future, further study in this area will be worthy of being developed.

Our results imply that green bond issuance is not merely virtue signaling in relation to environmental protection and sustainable development but can produce significant economic and environmental benefits. As the quantity of maturing green bonds increases, the importance of green bonds in improving companies' profitability, operational performance, and innovation capacity will be expected to emerge. To cater to this trend, China's green bond market requires continuous improvement, and green bond standards should be strictly implemented to accelerate the alignment of China's market with international community expectations. In terms of information disclosure, external supervision should involve in the theoretical design of green bonds and related trading systems to maintain the health of green projects. It is worthy of further research on information disclosure in ESG investment.

## Data Availability Statement

Publicly available datasets were analyzed in this study. This data can be found here: https://www.wind.com.cn.

## Author Contributions

SW: conceptualization, formal analysis, investigation, and writing-original draft. SW and DW: writing-review and editing. DW: funding acquisition. All authors have read and agreed to the published version of the manuscript.

## Funding

This research was funded by the National Natural Science Foundation of China (Grant Number: 71974201) and Capital University of Economics and Business (Grant Number: QNTD202005).

## Conflict of Interest

The authors declare that the research was conducted in the absence of any commercial or financial relationships that could be construed as a potential conflict of interest.

## Publisher's Note

All claims expressed in this article are solely those of the authors and do not necessarily represent those of their affiliated organizations, or those of the publisher, the editors and the reviewers. Any product that may be evaluated in this article, or claim that may be made by its manufacturer, is not guaranteed or endorsed by the publisher.

## Footnotes

^1^According to the company announcements, the Huazheng ESG evaluation system is based on the core connotation and development experience of ESG, combined with the actual situation of China's domestic market, to build a three-tier indicator system from top to bottom. Specifically, it includes 3 primary indicators, 14 secondary indicators, 26 tertiary indicators and over 130 underlying data indicators. The rating scope covers all A-share listed companies, and the rating is presented in the form of a total score of 100, with a corresponding rating of “AAA-C” in nine grades.
